# Does dual task training improve walking performance of older adults with concern of falling?

**DOI:** 10.1186/s12877-017-0610-5

**Published:** 2017-09-11

**Authors:** B. Wollesen, S. Schulz, L. Seydell, K. Delbaere

**Affiliations:** 1 Human Movement Science, University of Hamburg, Mollerstr. 10, Turmweg, 20148 Hamburg, Germany; 2 Department of Health Science, Neuroscience Research Australia, University of New South Wales, Margarete Ainsworth Building, Barker Street, Randwick, Sydney, NSW 2031 Australia

**Keywords:** Physical exercise, Fear of falling, Gait, Accidental falls

## Abstract

**Background:**

Older adults with concerns of falling show decrements of gait stability under single (ST) and dual task (DT) conditions.

To compare the effects of a DT training integrating task managing strategies for independent living older adults with and without concern about falling (CoF) to a non-training control group on walking performance under ST and DT conditions.

**Methods:**

Single center parallel group single blind randomized controlled trial with group-based interventions (DT-managing balance training) compared to a control group (Ninety-five independent living older adults; 71.5 ± 5.2 years).

A progressive DT training (12 sessions; 60 min each; 12 weeks) including task-managing strategies was compared to a non-training control group. Setting: group based intervention for independent living elderly in a gym. ST and DT walking (visual verbal Stroop task) were measured on a treadmill. Gait parameters (step length, step width, and gait line) and cognitive performance while walking were compared with a 2x2x2 Repeated Measures Analyses of Variance.

**Results:**

Participants in the intervention group showed an increased step length under ST and DT conditions following the intervention, for both people with and without CoF compared to their respective control groups. Foot rolling movement and cognitive performance while walking however only improved in participants without CoF.

**Conclusions:**

The results showed that DT managing training can improve walking performance under ST and DT conditions in people with and without CoF. Additional treatment to directly address CoF, such as cognitive behavioural therapy, should be considered to further improve the cautious gait pattern (as evidenced by reduced foot rolling movements).

**Trial registration:**

The study was retrospectively registered in the German Clinical Trials Register (DRKS; Identification number DRKS00012382, 11.05.2017).

## Background

Falls pose a major threat to the well-being and quality of life of older people. Falls can result in fractures and other injuries, disability and concern about falling (CoF), which trigger a decline in physical function and walking performance [1–3] and increase an individual’s risk of future falls [4, 5]. CoF is very common for independent-living older adults, with prevalence rates often exceeding those of falls themselves [6]. About half of older adults express some level of CoF, and women are more commonly affected than men [7].

Previous research [8] has demonstrated that CoF can induce gait adaptations, by manipulating the environment in a way that exacerbates the potential consequences of a fall. These experimental studies have suggested that CoF decreases walking speed and step length, and increases double support time. It is now well-accepted that walking is not just a rhythmic and automated process, but also demands attention [9]. These demands increase with age, and with the complexity of the task being performed. More specifically, the ability to inhibit inappropriate responses and selectively attend to relevant environmental features while suppressing other inputs [10]. Since most daily life activities include some level of dual-tasking, these executive functioning skills (i.e. inhibitory skills) are required. It has been proposed that people with higher levels of CoF cannot inhibit or ignore irrelevant information of the environment in the process of balance control. Therefore, during the cognitive process the CoF competes for the limited resources of attentional focus to maintain balance control [11], resulting in an increased gait variability, instability and fall risk. For single task (ST) walking performance, a meta-analysis by Ayoubi et al. [12] revealed significant effects of CoF expressed in increased gait variability. Under DT conditions, a study by Donoghue et al. [13] found reduced gait speed and step length, especially for older persons who reduced their daily physical activity due to their CoF.

Systematic review evidence [14] has demonstrated that DT training studies can improve DT walking performance (cadence, gait variability, walking speed, foot rolling [15, 16]). It has been suggested that better transfer effects can be achieved when DT training includes task managing strategies like task-switching training (switch of attention from one task to another [17]) or task prioritization training (focus on one of the tasks [18]). These aspects have not been integrated to intervention programs for older people with CoF, yet. Hence, it is not clear whether older adults with CoF benefit from this kind of DT training due to the additional cognitive efforts, which affect the attentional resources of this target group [10] in DT conditions. Therefore, the study aim was to examine whether a progressive DT balance-training intervention with a focus on task managing strategies (task switching and task prioritization) could improve DT walking performance in older adults with and without CoF. The hypothesis is that DT training effects with task managing strategies are beneficial to those participants with CoF because they address the cognitive skills needed for walking and dual-task walking.

### Aim

The primary aim of this study was to compare the effects of a DT training integrating task managing strategies for independent living older adults with and without concern about falling to a non-training control group on walking performance under ST and DT conditions.

## Methods

The study was retrospectively registered in the German Clinical Trials Register *(DRKS;* Identification number DRKS00012382, 11.05.2017).

### Design

A single blind randomized controlled trial investigated the effect of a DT group-based intervention on DT walking after 12 weeks. The study was approved by the ethics committee of the Hamburg Chamber of Physicians (registration number PV4376). All participants were informed about the study goals, and signed informed consent according to the Declaration of Helsinki. The setting was a group-based training (up to 15 persons in each training group) for independent elderly in a gym.

### Participants and recruitment

**Fig. 1:**
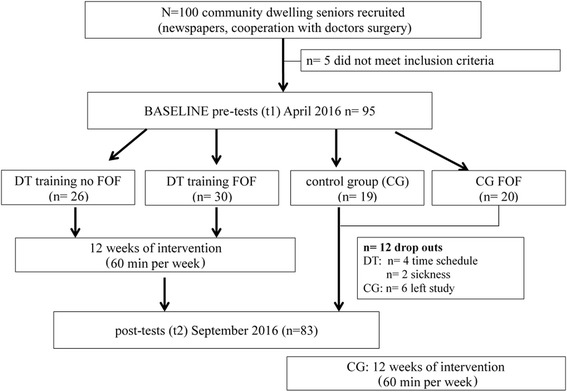
shows the study design and participant flow

A total number of 100 participants were recruited through advertisements in local newspapers. The inclusion criteria were: independent-living; age 65–80 years, able to walk without a walking aid and capable of attending the group training. Exclusion criteria were: acute or chronical diseases with a documented influence on balance control (e.g. Parkinson’s Disease; Diabetes Mellitus); cognitive impairment (Mini Mental Status Exam (MMSE) of less than 25); or participation in other exercise programs that could potentially confound the primary outcome. Figure 1 shows the study design and participant flow.

### Randomisation

The randomisation sequence was determined using a web-based program (http://www.randomizer.org) and which was conducted by a person not involved in the study. Randomisation was stratified by the Short Physical Performance Battery (SPPB) [19], Falls Efficacy Scale international (FES-I) [20], sex and age (± 2 years). Participants were randomized in a DT-managing balance training and a control group (cf. Fig. 1).

### Description of the intervention

#### DT-training

The group-based *DT* training program was conducted for twelve weeks, during one 60 min session per week, including 8 to 15 participants per group. The group session was instructed by specialized DT trainers in a gymnasium [21]. The trainers were specialized for the regime of the DT training. They are physical therapist with additional background of human movement sciences or human movement scientists with additional licenses for older aged participants and falls prevention.

During the first 6 weeks, the program focused on daily situations which are commonly associated with an increased fall risk and included mostly challenging over ground walking exercises (brisk walking, start-stop exercises, walking with sidesteps, walking with turns, walking while negotiating tripping hazards [22]). To further increase difficulty levels, additional challenges were added to the walking exercises: faster speed, reduced visual input (e.g. looking up to the sky or closing their eyes), reduced proprioceptive input (e.g. walking on compliant surfaces), and reduced area of support. During these walking exercises, participants were also exposed to a variety of cognitive tasks, designed to challenge their focus of attention (e.g. reacting to signs for changing directions or following rhythms introduced by the instructor). In addition to physical and visual modifications (speed, visual input) task managing strategies targeting inhibitory skills and task prioritization were introduced (i.e., prioritization of the motor task, the cognitive task or both tasks at the same time).

During the last 6 weeks, the program focused more on task prioritization, task-switching and transfer into daily situations. The tasks became more complex and included visio-spatial and executive function tasks (e.g. reaction on signs that say turn right but the instruction was to turn left). Additionally, all tasks from the first phase were trained under DT conditions combined with precision tasks, time pressure, task prioritization and task switching. Daily situations were imitated (e.g. carrying shopping bags in a crowd of people while avoiding obstacles or reacting on signs while shifting around other people) to practice task managing strategies.

The instructors explained all exercises with additional verbal feedback to improve ask performance. The importance of each exercise was explained in the context of fall prevention (education, increasing knowledge).

The training protocol was standardized and could be repeated by all participant similarly.

#### Control group

The control group participants did not receive any exercises for twelve weeks and carried on with their usual activities.

### Primary outcome measure

ST and DT walking performance was assessed during a 30-s walking test at self-selected constant speed on a treadmill (h/p/cosmos, Zebris; Isny, Germany: FDM-T). Before the test session the participants practiced treadmill walking for about five minutes in order to become familiar with the ST and DT walking task until they felt comfortable. A staircase method was used with going up to a certain level of comfortable walking speed and increasing and decreasing from that point until comfortable pace is achieved afterwards. Gait parameters such as step length (cm), step width (cm), and gait line, which describes the length of the foot rolling movements (cm) were measured as main outcome parameters. Our training regime focused mainly on the gait quality and associated the kinematic parameters. Since there is a controversial discussion about gait variability, for this study we focused on the reported gait parameters, which have been shown to be important walking variables as well [21].

A visual-verbal Stroop test with 30 events of incongruent coloured words (e.g. the word “blue” presented in a yellow font) was added to the walking test as a cognitive DT. Stimuli were projected onto a white wall two meters in front of the participants. Participants were asked to name the colour of the font letters word and inhibit reading the word. The time interval between word insertions randomly varied between 0.8 and 1.2 ms to avoid rhythm. A randomized process distributed three out of four different versions of the Stroop test to the participants (1. familiarization while sitting, 2. ST standing, 3. DT combined with treadmill walking) were presented to the participants. All tests were recorded as a Stroop video including the verbal responses to the visualised colour word on the screen. The number of correct answers was analysed.

### Other measures

Demographics, anthropometric data and comorbidities were assessed at baseline with a standardised questionnaire. Health-related quality of life was examined with the Short Form −12 questionnaire (SF 12 [23]).

CoF and fall risk were assessed as secondary outcome measures at baseline and after 12 weeks. The FES-I was used to examine concern about falling during 16 daily activities. The 16 items are rated as not at all concerned (1) to very concerned (4). Higher scores are indicative of greater concerns of falling. Participants with FES-I scores of 20 or higher were categorized into the higher CoF [20]. Intervention groups were stratified by the SPBB, due to previously established links between SPPB and kinematic parameters of walking [13, 21]. The SPPB was used as a control variable for the randomization process based on tests of static balance, walking speed over 4 m (at normal pace), and the five times chair stand test. Each test score is categorized from 1 (worst) to 4 (best); the overall sum is then used to create a SPPB summary performance scale.

### Sample size

A priori sample size calculation (G*power 3.1., ANOVA: Repeated measures within factors; f = 0.20; alpha error probability = 0.05; power = 0.80; number of groups = 2 × 2; number of measurements = 2) calculated a total number of 76 participants. With an anticipated dropout rate of 20%, the recruitment aimed for 92 participants.

### Statistical analysis

Twelve participants (six in the DT training group and six in the control group) did not have data available after 12 weeks (cf. Fig. 1). The main causes for drop out in the DT training group were problems with the time schedule (e.g. holidays *n* = 4) and illness (*n* = 2). Six participants of the control group left the study because of their group allocation. Repeated measures ANOVA were used to determine the intervention effect on the primary outcome at follow-up. Three-way (comparing 2 × 2 × 2 groups) repeated measures ANOVAs were used for each outcome variable (e.g. step length, step width, FES-I). Analyses were performed with SPSS version 22 (IBM statistics Armonk, NY). Main effects for time (pre/post), group (intervention/control) and CoF (low = FES-*I* < 20 /high FES-I 20 and above) were reported, as well as between-subject effects for the groups (Group x time x CoF). Significance level was set at a two-sided α of 5%; normal distribution was assessed using the Kolmogorow-Smirnow test. Variables were transformed as required to ensure statistical assumptions were met. Effect sizes are given as partial eta squares (η_p_^2^; small effect η_p_^2^ ≥ 0.08, medium effect η_p_^2^ ≥ 0.20, and η_p_^2^ ≥ 0.32 large effect, [24]. Bonferroni correction was applied to post-hoc comparisons.

## Results

Table 1 gives an overview of the main characteristics (*N* = 95) of the analysed participants.

**Table 1:** Mean (SD) or Number (%) of the Groups for the Demographic Characteristics of *N* = 95 Participants at baseline

Characteristics	Intervention with FES-*I* < 20 (*n* = 26)	Intervention with FES-*I* > 20 (*n* = 30)	Control group with FES-I < 20 (*n* = 19)	Control group with FES-I > 20 (*n* = 20)
Age (yr)	72.2 (4.6)	69.8 (5.7)	72.9 (4.4)	72.7 (5.3)
Females, number (%)	16 (61.5%)	28 (86.7%)	12 (63.2%)	17 (85%)
Height (cm) females	162.9 (5.4)	165.7 (6.6)	162.5 (9.6)	164.2 (5.6)
Height (cm) males	177.4 (9.7)	178.1 (3.0)	182.8 (5.5)	171.5 (6.2)
Weight (kg) females	64.3 (12.3)	72.3 (11.0)	70.6 (10.7)	73.8 (12.1)
Weight (kg) males	85.3 (15.7)	84.3 (13.7)	88.5 (5.2)	79.0 (20.1)
BMI females (kg/m^2^)	24.3 (3.7)	25.9 (3.5)	26.7 (3.5)	27.2 (3.6)
BMI males (kg/m^2^)	27.4 (2.9)	26.1 (4.2)	26.5 (1.)0	28.8 (2.5)
SPPB (score out of 12)	11.2 (0.9)	10.8 (1.4)	10.9 (1.3)	10.8 (1.0)
Walking speed (m/s)	4.49 (0.8)	4.25 (0.9)	4.84 (0.7)	4.0 (1.2)
Physical Problems (number)	2.4 (2)	2.5 (2)	2.5 (2)	3.1 (3)
Chronic diseases (number of participants)	8	15	7	9
Medications (number of participants)	16	19	9	13
FES-I (score out of 64)	17.9 (2.6)	23.4 (2.9)	17.6 (.9)	23.8 (3.4)
SF 12 physical(Reference score age group 37.76 ± 12.27)	46.84 (8.5)	47.27 (9.1)	47.80 (9.1)	48.41 (9.2)
SF12 mental (Reference score age group 50.24 ± 10.81)	49.65 (6.7)	51.65 (9.4)	52.10 (9.4)	53.20 (7.9)

*BMI* Body Mass Index, *SPPB* Short physical performance battery, *FES-I* Falls Efficacy Scale International, *SF12* Short Form −12 questionnaire

No significant group differences were found for demographic, gender and health-related data.

### Main effect of the intervention on participants with and without CoF

Table 2 shows all results of the three-way ANOVA. It shows significant effects of time - between pre to post measurements - for all examined gait parameters independent of the group allocation. The time × group interaction effects demonstrated a greater improvement for step length and gait line under ST and DT conditions of the intervention groups (with and without CoF). Post-hoc comparisons revealed an increased step length for both feet and gait line for the right foot (trend for left foot) in the intervention group (*p* < 0.001). In the DT conditions, there was also a time × group effect for the FES-I and DT walking test, as demonstrated by a reduced FES-I score (*p* < 0.001) and number of errors during the DT Stroop walking test in the intervention group (*p* < 0.05).

**Table 2:** Comparison of primary outcomes in the intervention and control groups (*N* = 83)

		Intervention no CoF (n = 20)		Intervention CoF (n = 30)		Control no CoF (*n* = 15)		Control CoF (*n* = 18)		Time (pre-post)		Group x time		Group x time x CoF	
Gait variables		Pre	Post	Pre	Post	Pre	Post	Pre	Post	F	p _p_η^2^	F	P _p_η^2^	F	P _p_η^2^
Single-Task pre- post															
step width [cm]		11.62 (3.03)	10.09 (3.57)	12.44 (3.09)	11.97 (3.0)	10.87 (4.14)	10.19 (3.17.)	12.66 (3.17)	11.73 (3.72)	11.678	>0.001 0.129	0.855	0.468 0.031	1.498	0.225 0.019
step length [cm]	l	43.77 (8.72)	49.25 (7.16)	37.37 (12.34)	41.58 (13.67)	51.12 (7.54)	49.54 (8.32)	40.60 (13.22)	41.06 (12.21)	12.141	>0.001 0.133	6.603	>0.001 0.201	1.802	0.183 0.022
	r	43.27 (9.44)	48.63 (7.90)	37.58 (13.65)	41.71 (14.34)	51.77 (7.57)	49.55 (8.14)	38.74 (13.97)	39.54 (13.91)	10.533	>0.01 0.118	6.966	>0.001 0.209	2.941	0.090 0.036
gait-line [mm]	l	198.10 (57.05)	219.30 (38.39)	179.73 (38.70)	185.73 (35.23)	230.87 (41.68)	233.13 (46.37)	184.00 (47.73)	191.39 (42.99)	10.916	>0.001 .121	2.184	.096 .077	3.319	0.072 0.040
	r	193.40 (60.18)	220.85 (37.85)	182.68 (37.60)	191.17 (32.62)	232.68 (43.69)	234.87 (46.32)	186.06 (45.67)	192.90 (48.37)	14.959	>0.001 .159	3.570	.018 .119	4.112	0.046 0.049
Dual Task pre –post															
step width [cm]		10.77 (2.98)	10.25 (3.38)	12.37 (4.0)	12.24 (3.23)	11.0 (3.42)	10.32 (2.76)	12.95 (4.01)	12.59 (4.28)	.573	0.452 0.010	0.246	.783 .009	0.004	0.950 0.000
step length [cm]	l	44.35 (9.70)	50.09 (7.75)	38.75 (13.0)	41.63 (13.22)	50.45 (6.25)	44.39 (7.30)	41.26 (11.61)	41.24 (12.26)	9.230	>0.01 .105	5.246	>0.01 .166	1.666	0.201 0.021
	r	43.89 (10.20)	49.16 (8.39)	38.03 (13.78)	41.02 (14.07)	51.50 (5.49)	49.91 (7.54)	40.13 (14.01)	39.82 (13.79)	5.109	> 0.05 .061	4.603	>0.01 .149	1.597	0.210 0.020
gait-line [mm]	l	210.50 (42.58)	222.00 (36.34)	180.87 (43.92)	186.47 (37.80)	236.73 (40.05)	237.47 (45.66)	199.72 (39.99)	200.50 (35.57)	2.971	.089 .036	0.852	0.470 0.031	0.303	0.583 0.004
	r	207.70 (47.06)	222.10 (34.50)	186.50 (37.57)	194.13 (30.61)	237.80 (39.44)	236.00 (47.21)	203.79 (39.92)	200.39 (44.17)	2.950	.090 .036	2.802	> 0.05 .096	0.029	0.559 0.004
Secondary outcomes pre-post															
FES-I		17.05 (1.10)	17.00 (0.92)	23.73 (2.80)	21.80 (3.24)	17.60 (0.99)	17.60 (1.12)	23.83 (3.50)	23.66 (4.02)	9.822	0.002 0.111	5.435	0.002 0.171	4.471	0.038 0.054
SF12 physical		46.76 (8.80)	48.82 (6.88)	47.07 (9.40)	48.81 (9.01)	47.82 (9.17)	48.33 (7.22)	48.41 (9.14)	46.53 (10.18)	0.523	0.473 0.009	1.133	0.344 0.058	0.379	0.541 0.007
SF12 mental		49.46 (6.92)	51.98 (6.78)	51.21 (9.63)	53.58 (9.44)	52.98 (5.41)	56.58 (3.47)	53.20 (7.40)	54.53 (7.70)	13.513	0.001 0.197	0.377	0.770 0.020	0.626	0.432 0.001
SPPB		11.05 (0.94)	11.65 (0.75)	10.73 (1.48)	11.23 (1.04)	10.93 (1.28)	11.27 (0.96)	10.89 (0.96)	11.22 (1.06)	14.008	>0.001 0.151	0.298	0.827 0.011	0.045	0.833 0.001
Number of correct answers Stroop sitting (0–30)		27.3 (2.6)	28.1 (1.7)	26.9 (4.3)	28.2 (2.5)	28.7 (2.5)	25.3 (9.5)	25.1 (5.2)	25.6 (4.0)	0.096	0.758 0.002	2.009	0.123 0.097	2.115	0.151 0.036
Number of correct answers Stroop walking (0–30)		27.7 (2.7)	28.2 (1.8)	27.0 (3.9)	27.7 (2.4)	27.5 (3.8)	23.5 (11.1)	25.2 (3.9)	26.1 (3.5)	1.084	0.302 0.019	3.489	0.022 0.157	6.230	0.016 0.100

### Differentiating effect of the intervention in people with and without CoF

There were no strong time × group × CoF effects for ST or DT walking, with only a significant effect for the right foot gait line under ST conditions (Table 2). Participants without CoF in the intervention group showed greater increases for the gait-line of the right foot (*p* < 0.05). The FES-I decreased in people with CoF who had the intervention compared to the control group, and remained unchanged in the no CoF group. The cognitive performance while DT walking improved in people in the intervention group without CoF compared to the control group, with no effect in people with CoF (*p* < 0.05).

## Discussion

The present study examined whether a progressive DT balance-training intervention with a focus on task managing strategies (task switching and task prioritization) could improve DT walking performance in older adults with and without CoF. The results showed a significantly improved ST and DT walking performance for the intervention group regardless of CoF after 12 weeks of training. Improved walking performance was demonstrated through an increased step length and gait line. These common kinematic walking variables suggest an active use of the ankle joint and roll movements, which is an important strategy to compensate for gait impairment and maintain postural control while walking [25]. Gait improvements were evident for the participants of the intervention independent of CoF under both ST and DT conditions, similar to previous studies using similar DT interventions with task-managing strategies [15–18, 21].

The overall aim of this present study was to explore whether there was a disparate effect of our DT intervention in older people with CoF. In line with previous studies [12, 13], participants with CoF walked slower at baseline with a reduced overall walking performance compared to people without CoF, under both ST and DT conditions. However, while our DT training, improved step length, equally in both groups independently of CoF, our results show that the gait-line did not follow that same trend. This suggests that people with CoF still have more cautious gait pattern after the intervention with less foot rolling movements compared to the participants with no CoF [10, 26]. A recent Cochrane review has suggested that exercise can reduce CoF immediately following the intervention [27], however, there is less certainty about long-term effects. Our finding that foot rolling movement remain impaired following the intervention in people with CoF might be an explanation for a reduced effect of exercise for participants with CoF. Since the exercises did not address the CoF, additional psychological support might be required to fully address CoF and the underlying reason for the reduced DT performance e.g. Cognitive Behaviour Training (CBT) for people with CoF.

Our results also show that cognitive performance while walking did not improve in people with CoF, which also confirms that they might require additional support through strategies such as CBT to minimize the interference of CoF on the capacity to perform tasks requiring attentional resources. The resources allocation model by Kahnemann [28] suggests that a higher amount of attention is needed for motor and cognitive performance under DT conditions to manage both tasks similarly. If CoF competes for these attentional resources, walking will be more strongly affected by a DT in people with CoF. Following the task prioritization model by Yogev-Seligmann et al. [29] one might argue that the laboratory situation was not dangerous enough to switch from the cognitive focus to motor control. Therefore, future studies should include a more challenging walking task to control if this balance and task managing intervention is successful in a transfer to daily situations.

### Limitations

We would like to acknowledge some limitations regarding the study design. Despite our attempts to stratify participants based on fall risk and COF into control and intervention, our groups were not equal. The control group participants had a higher walking speed and increased step length. Furthermore, participants were allocated using a 2:1 ratio to intervention and control groups, respectively. This decision was guided by our previous experiences that participants are often displeased by a control group allocation. This combined with a high attrition rate in the control group due to group allocation, amplified the differences in group size further for the effectiveness analyses, which might have affected the representativeness of the sample. Moreover, we did not include a pre-post fall risk assessment or an over-ground gait assessment. The study was not powered to look at differential effects of certain tasks or dual-task cost. Additionally, one might argue that the participants trained over ground walking performance, whereas the test situation was done on a treadmill. To avoid confounding factors, we followed the recommendations by Wollesen, Rönnfeldt and Mattes [30]. However, there is some evidence that there are no kinetic and kinematic differences when over ground walking is compared to treadmill walking [31, 32]. Nevertheless, the measurement on the treadmill with fixed self-selected walking speed does not allow the analysis of improvements in walking speed. In addition, due to the small sample size dual-task costs could not be analysed sufficiently. Therefore, these aspects should be controlled in future studies.

## Conclusions

In conclusion, the present study demonstrated that our DT training intervention including task managing strategies was effective for both participants with and without CoF, by improving overall gait performance under ST and DT conditions. However, there was a disparate effect for people with CoF compared to people without CoF for the active foot rolling movements and cognitive performance while walking, where people with CoF showed less improvement following the intervention on both factors. To see long-term effects on CoF and gait performance, we suggest the addition of psychological strategies, such as CBT to DT training.

## Abbreviations

CBT: Cognitive Behaviour Training
CoF: Concerns of Falling
DT: Dual task
FES-I: Falls Efficacy Scale international
MMSE: Mini Mental Status Exam
SF 12: Short Form (12 questionnaire) SF 36 Health Survey
SPPB: Short Physical Performance Battery
ST: Single task
